# Hypoglossal nerve palsy after SARS-CoV-2 vaccination – report of two cases

**DOI:** 10.1186/s12883-022-02929-2

**Published:** 2022-11-09

**Authors:** J. Panholzer, L. Kellermair, C. Eggers

**Affiliations:** Department of Neurology, Kepler University Hospital, Linz, Austria

**Keywords:** Hypoglossal nerve palsy, SARS-CoV2 vaccine, Autoimmune disorder

## Abstract

**Background:**

SARS-CoV-2 vaccination is associated with an increased risk for Bell’s palsy and some other neurological disorders assumed to be of autoimmune origin. While facial nerve palsy is frequent and usually idiopathic, hypoglossal nerve palsy is rare, and a specific cause is almost always found. We firstly report two patients who developed isolated hypoglossal nerve palsy shortly after SARS-CoV-2 vaccination.

**Case presentation:**

Two otherwise healthy patients, a 49-year-old man and a 39-year-old woman, developed unilateral hypoglossal nerve palsy 10 and 7 days after the second SARS-CoV-2-vaccination (AstraZeneca and BioNTech/Pfizer), respectively. In both subjects, needle electromyography showed denervation and rarefication of motor units. CT, MRI, examination of blood and CSF as well as ENT exam were unremarkable. In both subjects symptoms gradually improved.

**Conclusion:**

Due to close temporal relationship, the absence of other etiologies, and spontaneous improvement we suspect the vaccination as the cause for hypoglossal nerve palsy in both patients. This is further supported by the rarity of isolated hypoglossal nerve palsies, especially in idiopathic cases. We suggest the addition of hypoglossal nerve palsy to the list of neurological injuries potentially caused by SARS-CoV-2 vaccination.

## Background

Vaccination-induced neurological disorders have long been recognized and are thought to be of autoimmune origin. SARS-CoV-2 vaccination has recently been shown to carry an increased risk for facial nerve palsy, Guillain-Barré syndrome and myasthenic disorders [1]. Facial nerve palsy is the most common cranial nerve palsy with an incidence of 7–40 cases per 100 000 persons per year, and its idiopathic form (Bell’s palsy) makes up for 60–75% of cases [2]. Hypoglossal nerve palsy, on the other hand, is rare. We first report two patients who developed isolated hypoglossal nerve palsy shortly after SARS-CoV-2 vaccination.

## Case presentation

**Fig. 1:**
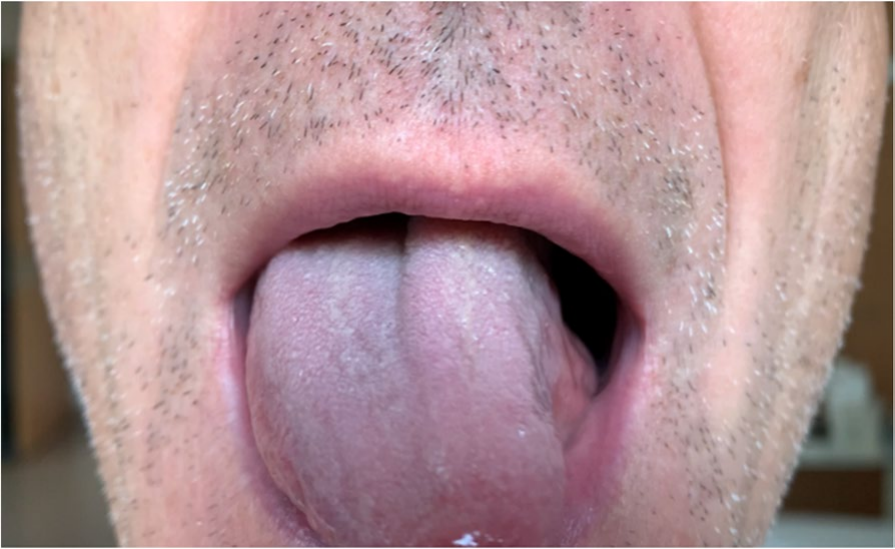
49-year-old man with left-sided hypoglossal nerve palsy after second SARS-CoV-2 vaccination. The palsy developed ten days after application of AstraZeneca vaccine and improved in the following weeks without specific treatment

Two otherwise healthy patients, a 49-year-old man and a 39-year-old woman, noted difficulties in moving chyme with their tongue. Symptoms occurred ten and seven days, respectively, after their second vaccination for SARS-Cov-2. The first patient received a vaccine from AstraZeneca, the second patient one from BioNTech/Pfizer. On examination there was marked left-sided deviation of the tongue in both patients (Fig. 1). In both subjects, needle electromyography of the left superior longitudinal muscle of the tongue showed fibrillation potentials, positive sharp waves and rarefication of motor units. Computed tomography, including the skull base, brain magnetic resonance imaging and ENT exam were unremarkable. Examination of blood and CSF including rheumatologic parameters, CSF cytology, culture, neurotropic viruses, serology, onconeural and antineuronal antibodies revealed no relevant abnormalities. Both subjects improved significantly over the following weeks without specific treatment.

An extensive literature research on PubMed with the broad search term “hypogloss* AND (paresis OR paralysis OR palsy)” revealed 467 cases with either clinically predominant or completely isolated hypoglossal nerve lesion. Only 68 of these (14%) were classified as idiopathic.

## Discussion and conclusions

As we found no other etiologies and due to close temporal relationship, we suspect SARS-CoV-2 vaccination as the cause for hypoglossal nerve palsy in both patients. This is supported by the rarity of isolated hypoglossal nerve palsies of whatever origin, even more so of idiopathic cases. Vaccination-related 12^th^ nerve injury is very rare. In the current literature only one case of a 12^th^ nerve palsy occurring shortly after influenza vaccination in a 7-month-old girl with cystic fibrosis is reported [3]. Autoimmune reactions such as antibodies cross-reacting with peripheral myelin proteins are thought to be an important mechanism for post-vaccination neurological sequelae [4]. A large case–control study found that SARS-CoV-2 vaccination conferred a significantly increased risk for Bell’s palsy, Guillain-Barré syndrome and myasthenic disorders, all of which are linked to autoimmune etiologies [1].

The risk for isolated cranial nerve palsies such as Bell’s palsy and for combined cranial nerve palsies such as Miller Fisher syndrome or Guillain–Barre syndrome is substantially higher after SARS-CoV-2 infection than after receiving SARS-CoV-2 vaccination [1]. There are a few reports of isolated hypoglossal nerve palsies and combined affection including other cranial nerves after COVID-19 [5, 6].

In conclusion, we suggest the possibility of, albeit rare, SARS-CoV-2 vaccination-induced isolated hypoglossal nerve palsy. However, definitive proof of causality requires postvaccination surveillance programs of larger populations.

## Data Availability

Data will be provided by contacting Christian Eggers.

## Abbreviations

SARS-CoV-2: Severe acute respiratory syndrome coronavirus 2
ENT: Ears, nose, throat
CSF: Cerebrospinal fluid
